# Improving gene editing of CRISPR/Cas9 using the callus-specific promoter *pYCE1* in cassava (*Manihot esculenta* Crantz)

**DOI:** 10.3389/fpls.2025.1600438

**Published:** 2025-05-20

**Authors:** Yuanchao Li, Ruxue Bao, Mengtao Li, Changying Zeng, Haojie Yang, Yuan Yao, Youzhi Li, Wenquan Wang, Xin Chen

**Affiliations:** ^1^ State Key Laboratory for Conservation and Utilization of Subtropical Agro-bioresources/College of Life Science and Technology, Guangxi University, Nanning, Guangxi, China; ^2^ National Key Laboratory for Tropical Crop Breeding, Institute of Tropical Bioscience and Biotechnology, Chinese Academy of Tropical Agricultural Sciences, Sanya/Haikou, Hainan, China; ^3^ Institute of Tropical Agriculture and Forestry, Hainan University, Haikou, China; ^4^ Key Laboratory for Biology and Genetic Resources of Tropical Crops of Hainan Province, Hainan Institute for Tropical Agricultural Resources, Haikou, Hainan, China; ^5^ Sanya Research Institute, Chinese Academy of Tropical Agricultural Sciences, Sanya Hainan, China

**Keywords:** cassava, callus, promoter, gene editing, target

## Abstract

Previous studies have demonstrated that an appropriate promoter can drive *Cas9* transcription in the CRISPR/Cas9 system, which improves the efficiency of gene editing. Here, we identified and characterized callus-specific promoters to enhance gene editing efficiency in cassava. From the transcriptome data of 11 cassava tissues, the gene named *YCE1* was identified to exhibit callus-specific expression. Its promoter (*pYCE1*) could efficiently and specifically drive *EGFP* transcription in callus tissues. Given that friable embryogenic callus (FECs) is the recipient for genetic transformation in cassava, we replaced the original *35S* promoter with *pYCE1* to drive *Cas9* transcription for improving the CRISPR/Cas9 gene editing system. In single-gene editing, the mutation rate was significantly increased, which reached an overall mutation rate of 95.24% and a homozygous mutation rate of 52.38%, compared with 62.07% and 37.93% with the *35S* promoter, respectively. Furthermore, achieving a dual-gene homozygous mutation rate of 64.71% in dual-gene editing demonstrated the high efficiency of *pYCE1* in the gene editing application for cassava. These results underscore the potential of *pYCE1* to enhance gene editing efficiency in the CRISPR/Cas9 system of cassava. This approach paves the way for advanced gene function research and genetic breeding in cassava.

## 1 Introduction

The CRISPR/Cas system was originally discovered in *Archaea*, where it functions as a defense mechanism against invading foreign DNA by precisely cleaving the target DNA (Marraffini, 2015; Sorek et al., 2008). In 2012, this system was engineered to allow Cas9 to be directed by a single-guide RNA (sgRNA) to cut specific DNA sequences, which resulted in DNA double-strand breaks (DSBs) (Jinek et al., 2012). Subsequently, the CRISPR/Cas9 system was introduced into human and mouse cells, where it successfully achieved targeted gene mutation (Cong et al., 2013; Mali et al., 2013). The CRISPR/Cas9 gene editing system is primarily composed of two components: Cas9 nuclease and sgRNA. Cas9 recognizes the protospacer adjacent motif (PAM) sequence (NGG) in the genome and performs cleavage on the target DNA. sgRNA is a synthetic noncoding RNA that combines the functions of crRNA and tracrRNA, which guides Cas9 to specific target sites for cleavage (Hwang et al., 2013; Mali et al., 2013). Currently, by designing specific sgRNA, the CRISPR/Cas9 gene editing system can guide Cas9 to cleave specific genomic DNA sequences accurately (Feng et al., 2013; Shan et al., 2013). This approach, in combination with DNA repair mechanisms such as non-homologous end joining or homologous recombination, enables precise gene insertions, knockouts, or replacements (Li et al., 2016; Lu et al., 2020; Wang et al., 2017). The advancement of gene editing technologies has facilitated the development of new CRISPR/Cas systems, such as CRISPR/Cpf1, which recognizes a different PAM sequence (TTTN) and generates sticky ends upon cutting the target sequence. These properties of Cpf1 allow it to target different sites within the genome, which provides more options for gene editing (Fonfara et al., 2016; Zetsche et al., 2015). Moreover, leveraging the DNA-binding capability of the CRISPR/Cas9 system, catalytically inactive Cas9 variants (dCas9) have been fused with cytidine deaminase, adenine deaminase, and reverse transcriptase to develop new editing systems, such as base editing and prime editing. Base editing involves precise modifications at specific nucleotide position without inducing DSBs (Hua et al., 2019; Komor et al., 2016; Liu et al., 2021; Yang et al., 2023). Prime editing enables various genomic edits, such as base substitution, insertion, and deletion, without introducing DSBs or requiring donor DNA at the target site. This approach significantly reduces unnecessary insertions and deletions (indels) and off-target effects, which enhances editing precision (Anzalone et al., 2019; Chen et al., 2021; Ponnienselvan et al., 2023).

The efficiency of the CRISPR/Cas9 system is primarily influenced by the structure of the sgRNA and the expression abundances of *Cas9* and sgRNA (Char et al., 2017; Zheng et al., 2020; Zhou et al., 2018). Using online tools such as CRISPR-P2.0 to carefully select specific target sequences within the sgRNA, the risk of off-target effects can be minimized, and the gene editing efficiency can be significantly enhanced (Liu et al., 2017). In the CRISPR/Cas9 system, promoters play a crucial role given that they directly impact the transcription activities of *Cas9* and sgRNA. Promoters of RNA polymerase III-type small nuclear RNA genes, such as *U3* and *U6*, are commonly used to drive sgRNA transcription, with *U3* being specific for monocots and *U6* for dicots (Ma et al., 2015). The constitutive promoter *35S* is often used to drive *Cas9* transcription, which ensures widespread accumulation of Cas9 protein in transgenic plants. However, this approach results in a low homozygous mutation rate (Odipio et al., 2017). Moreover, if the editing is incomplete or generates heterozygous mutation types, then sexual or asexual propagation can result in variations in the mutation types among progeny (Feng et al., 2013; Odipio et al., 2017). Replacing *35S* with tissue-specific promoters such as *YAO*, *EC1.2*, and *SPL* has demonstrated high-efficiency gene editing in T_0_ generation of transgenic plants, with a rate of 80.9%–100% (Mao et al., 2016; Wang et al., 2015; Yan et al., 2015). Similarly, using the callus-specific expression promoter of the *ZmDMC1* gene to enhance *Cas9* transcription has increased the editing rate in T_0_ maize transgenic lines to 85.0%, with a homozygous editing rate of 66.0%; this rate is significantly higher than that of the *35S* and *UBQ* promoters (Feng et al., 2018).

Cassava is a crucial food crop in tropical and subtropical regions, and CRISPR/Cas9 technology has been extensively utilized due to its gene function validation and genetic improvement (Dong et al., 2023; Zhang, 2022). In recent years, gene editing mutants, such as *meptst1*, *megbss1*, and *mesbe2* (Bull et al., 2018; Luo et al., 2022), have been successfully developed, which resulted in the creation of novel lines with different amylose/amylopectin ratios in storage root starches. In addition, editing *MeCYP79D1* and *MeCYP79D2* has significantly reduced the cyanogenic glycoside content in cassava leaves and storage roots (Gomez et al., 2023; Juma et al., 2022), while editing *MenCBP-1*/*-2* and *MeSWEET10a* has enhanced resistance to cassava brown streak virus and cassava bacterial blight (Elliott et al., 2024; Gomez et al., 2019; Wang et al., 2024). Although *35S*::Cas9 can be used for targeted gene editing in cassava, its resulting homozygous mutation rate is low, which usually does not exceed 33.34% (Odipio et al., 2017). Furthermore, the highly heterozygous nature of cassava genome and the lengthy transformation cycle cause difficulty for the desired traits to stably manifest in heterozygous mutants (Utsumi et al., 2017). Therefore, improving the rate of homozygous mutant production is critical. Drawing from gene editing optimization strategies in other species, cassava also requires the use of endogenous promoters to increase *Cas9* transcription. Given that cassava transformation is mediated by *Agrobacterium* infection of friable embryogenic callus (FECs) (Nyaboga et al., 2015; Zainuddin et al., 2012), callus-specific promoters are needed to drive *Cas9* transcription efficiently. This process enhances homozygous mutation rate and minimizes off-target effects by reducing Cas9 protein accumulation in other tissues.

In this study, we identified and cloned the promoter of *YCE1* gene (Manes.18G120800), which is specifically and highly expressed in cassava callus. The promoter of *YCE1* replaced the commonly used *35S* to drive *Cas9* transcription in the CRISPR/Cas9 system. Subsequently, we conducted single- and dual-gene editing experiments in cassava. Our results demonstrated that the use of *pYCE1*::*Cas9* not only achieved high-efficiency gene editing but also successfully achieved efficient dual-gene homozygous mutation.

## 2 Materials and methods

### 2.1 Plant materials and growth conditions

In this study, the cassava cultivar SC8 was selected and grown in the experimental field in Haikou, Hainan Province. Transgenic plants were cultured in a greenhouse under growth conditions of 16 h light and 8 h dark, with a constant temperature of 28°C. The FECs used for genetic transformation were induced from the SC8 cultivar following previous study (Wang et al., 2022).

### 2.2 RNA extraction and real-time quantitative PCR

Samples of leaf, midvein, petiole, lateral bud, stem, fibrous root, and storage root were collected from cassava plants grown for 120 d in the experimental field, and callus were induced from SC8. Total RNA from each sample was extracted using the Plant Total RNA Isolation Kit Plus (Foregene, Chengdu, China) following the instructions of the manufacturer. Subsequently, 4 µg total RNA was reverse transcribed into cDNA using the PrimeScriptTM IV 1st strand cDNA Synthesis Mix (Takara, Dalian, China) according to the instructions of the manufacturer. The expression levels of target genes were analyzed using SYBR^®^ Premix Ex TaqTM II (Takara, Dalian, China) reagents and the Roche LightCyclerTM real-time PCR system as per the instructions of the manufacturer. *MeActin* (Manes.12G150500) was used as an internal reference to calculate the relative expression levels of target genes. Primers used for RT-qPCR are listed in **Supplementary Table S2**.

### 2.3 Cloning of promoters and *cis*-element analysis

Using the genomic DNA of SC8 as a template, promoter sequences of*YCE1* and *YCE2* genes were amplified using PrimerSTAR Max Mix (Takara, Dalian, China) and specific primers pYCE1-F/pYCE1-R and pYCE2-F/pYCE2-R (**Supplementary Table S2**). The PCR reaction volume comprised 10.0 μL 2×PrimerSTAR Max Mix, 0.5 μL of each forward and reverse primer (10 μmol/L), 1.0 μL genomic DNA (100 ng/μL), and ddH_2_O to a final volume of 20.0 μL. The PCR amplification conditions were as follows: initial denaturation at 94°C for 3 min, followed by 30 cycles of 98°C for 30 s, 60°C for 10 s, and 72°C for 1 min, with a final extension at 72°C for 5 min, and storage at 16°C. The PCR products were separated by 1% agarose gel electrophoresis, and the target DNA fragments were recovered using a kit (Magen, Guangzhou, China). The recovered *pYCE1* and *pYCE2* DNA fragments were ligated into a T-vector and then transformed into *DH5α* competent cells. Positive clones were selected and sent for sequencing. After sequencing confirmation of the cloned promoter sequences, *cis*-elements within the *pYCE1* and *pYCE2* regions were assessed using the online tool PlantCARE (https://bioinformatics.psb.ugent.be/webtools/plantcare/html/) (Lescot et al., 2002).

### 2.4 Vector construction

The sequenced-verified *pYCE1*-T and *pYCE2*-T plasmids were used as templates to construct the *pYCE1*::*EGFP* and *pYCE2*::*EGFP* vectors. Specific primers pYCE1-GFPF/pYCE1-GFPR and pYCE2-GFPF/pYCE2-GFPR were utilized to amplify the promoter sequences of *YCE1* and *YCE2* by PCR, respectively. These promoter sequences were subsequently inserted upstream of *EGFP* gene in the binary vector *pCAMBIA-G1300* at the *Pst* I/*Sal* I sites to replace the original *35S.* To utilize *pYCE1* in the CRISP/Cas9 gene editing system, the *pYCE1* fragment was amplified using the primers pYCE1-casF/pYCE1-casR and inserted upstream of the *Cas9* in the *pCAMBIA1301-Cas9* vector at the *Hind* III/*Nco* I sites to replace *35S* for driving *Cas9* expression. Gene editing vectors were constructed by selecting high-scoring, off-target free editing sites through the online platform CRISPR P2.0 (Liu et al., 2017). The construction followed the protocol described by previous study (Xie et al., 2015).

### 2.5 Genetic transformation and plant tissue culture

This study used the cassava variety SC8 to induce FECs. The experimental steps were as follows: first, 1 cm stem segments with lateral buds were excised from *in vitro* plants grown for 4 weeks to 8 weeks. These segments were placed on CAM solid medium (MS + 10 mg/L 6-Benzyladenine) and incubated in darkness at 28°C for 4 d to promote lateral bud enlargement. The enlarged buds were then excised and transferred to CIM solid medium (MS + 1 mg/L Picloram), where they were cultured in darkness at 28°C for 2 weeks to induce somatic embryo formation. Finally, the somatic embryos were transferred to GD solid medium (with 1 mg/L Picloram) for further culture to promote the formation of FECs (Utsumi et al., 2017; Wang et al., 2022; Zainuddin et al., 2012).

Genetic transformation of cassava was performed via the *Agrobacterium*-mediated method. The transformation vector was introduced into *Agrobacterium* strain *LBA4404* and cultured in LB medium (with Kanamycin and Rifampicin) at 30°C until the bacterial suspension reached OD_600_: 0.8–1.0. The suspension was washed twice with GD medium (with 1 mg/L Picloram, 250 μM Acetosyringone) and adjusted to OD_600_: 0.65. FECs were co-cultured with the bacterial suspension at 28°C and 50 rpm for 40 min. After co-cultivation, the FECs were transferred to GD medium (with 1 mg/L Picloram, 250 μM Acetosyringone) and incubated in the dark at 28°C for 3 d. Residual bacteria were removed by washing the FECs with GD medium containing 500 mg/L carbenicillin, followed by cultivation on GD solid medium (with 250 mg/L Carbenicillin) at 28°C for 3 weeks, with weekly medium replacement. Thereafter, the FECs were transferred to MSN medium (MS + 1 mg/L Naphthaleneacetic acid, 10 mg/L hygromycin) for cotyledon formation. They were further differentiated on CEM medium (MS + 0.4 mg/L 6-Benzyladenine, 10 mg/L Hygromycin). Mature transgenic cassava plants were obtained on MS solid medium (Wang et al., 2022).

### 2.6 Identification of transgenic positive plants

In this study, after the plants with the *EGFP* gene were successfully transformed, we first used a handheld laser emitter to observe EGFP expression in the root for preliminary identification of positive plants. Subsequently, the transgenic positive plants were further confirmed by PCR using specific primers GFP-F and GFP-R. For the positive plants transformed with the CRISPR/Cas9 editing vector, PCR identification was similarly conducted using the specific primers Cas9-F and Cas9-R to confirm the successful integration and functional expression of the CRISPR/Cas9 system.

### 2.7 Sanger sequencing and Hi-Tom sequencing analysis

In this study, genomic DNA was extracted from the verified positive plants by using DNA from wild-type plants as a control. Specific primers were first used to amplify DNA fragments covering the target gene editing sites, and the PCR products were assessed using 1.0% agarose gel electrophoresis. After the PCR products were recovered, sanger sequencing was performed to detect potential gene mutations. The Hi-Tom technique was applied to analyze the amplified DNA fragments for further accurately identifying the types of editing events and their editing efficiencies in the positive plants (Liu et al., 2017).

### 2.8 DAB staining

The DAB (3,3’-diaminobenzidine) histochemical staining method was employed to detect the reactive oxygen species content in cassava leaves. Based on the method described by previous study (Daudi and O’Brien, 2012), 1.0 mg/ml DAB solution was prepared and adjusted pH to 3.0. Cassava leaves were immersed in the DAB solution and stained with shaking at 80–100 rpm for 6 h. After staining, a 75% ethanol solution was used to remove chlorophyll from the leaves, which allowed for a clearer observation of staining. The processed leaves were photographed against a white background for subsequent analysis and comparison.

## 3 Result

### 3.1 Screening and identification of callus-specific expressed genes in cassava

**Figure 1:**
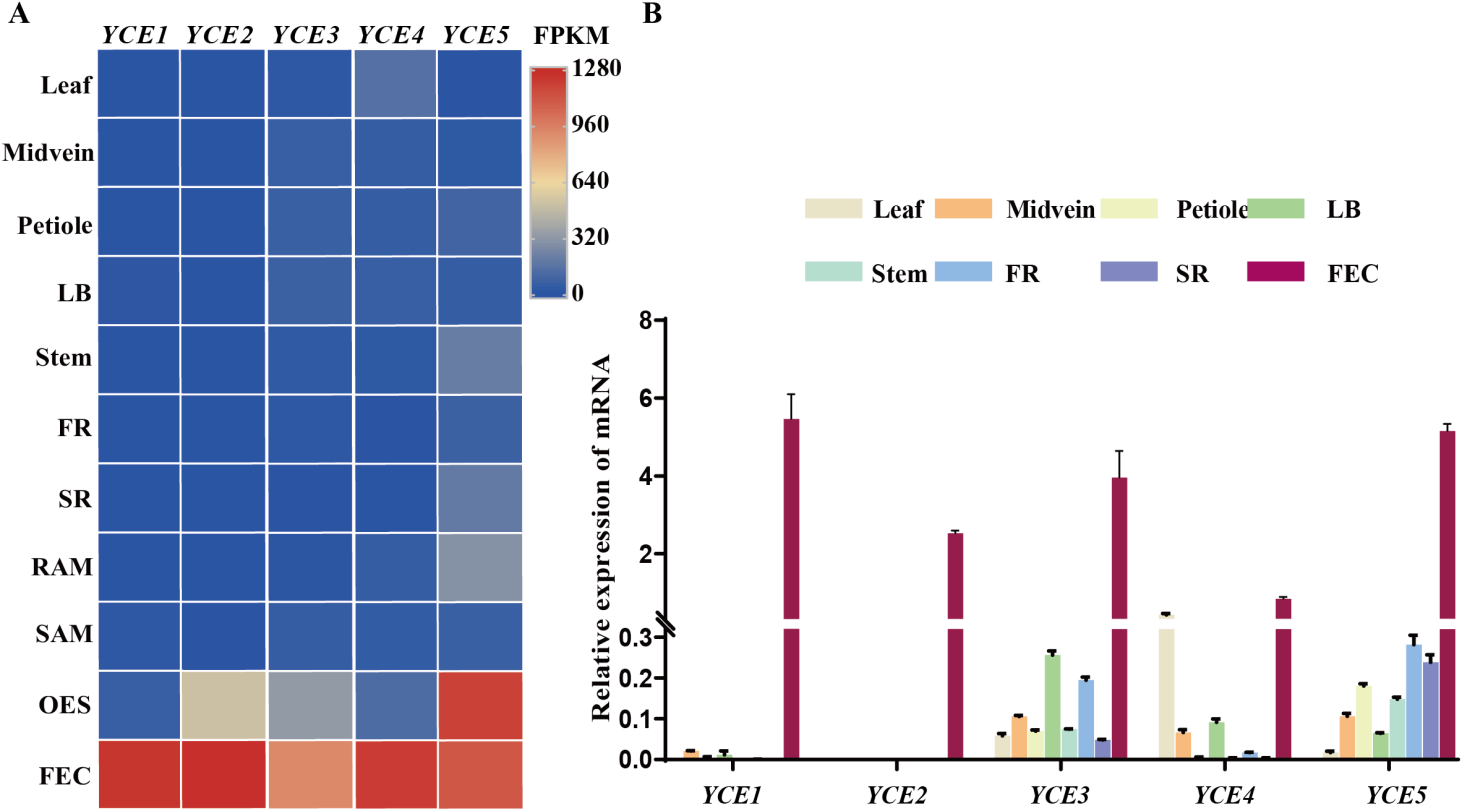
Identification of callus-specific expressed genes. **(A)** Candidate genes with high expression levels in callus tissues. LB, lateral bud; FR, fibrous root; SR, storage root; RAM, root apical meristem; SAM, shoot apical meristem; OES, organized embryogenic structures; FEC, friable embryogenic callus. **(B)** Validation of callus-specific highly expressed genes by RT-qPCR.

In the published transcriptome data of 11 different cassava tissues (Wilson et al., 2017), we preliminarily identified five genes that are highly expressed in FEC tissue: Manes.18G120800 (*YCE1*), Manes.08G121800 (*YCE2*), Manes.02G200000 (*YCE3*), Manes.16G137700 (*YCE4*), and Manes.18G087400 (*YCE5*) (**Figure 1A**). Samples of various cassava tissues were collected to perform a real-time qPCR assay for further confirming the callus-specific expression of the five candidate genes. The results showed that *YCE1* and *YCE2* were nearly silenced in leaf, midvein, petiole, lateral buds, stem, fibrous root, and storage root. However, they were highly expressed in FECs, which indicated their callus-specific expression. On the contrary, although *YCE3*, *YCE4*, and *YCE5* were highly expressed in callus tissue, they were also expressed in other tissues; thus, they lacked callus specificity (**Figure 1B**).

### 3.2 Cloning the promoters of *YCE1* and *YCE2*

We cloned the promoters of *YCE1* and *YCE2* to further investigate their callus-specific high expression. The promoter sequences of two genes were retrieved from the JGI database and analyzed for cis-acting regulatory elements using the PlantCARE tool. Based on the distribution and characteristics of the identified elements, the promoter regions were extended to include additional upstream sequences for further analysis. Using genomic DNA from the cassava cultivar SC8 as a template, promoter sequences of *YCE1*(1250 bp) and *YCE2*(1063 bp) were amplified (**Supplementary Figure S1A**). Sequencing results revealed that the cloned promoters shared over 99.5% similarity to the cassava AM560 reference genome, which confirmed the successful cloning of *YCE1* and *YCE2* promoters. Further analysis of the *pYCE1* and *pYCE2* sequences showed that both contained typical eukaryotic RNA polymerase II binding sites, including the TATA-box and the enhancer *cis*-element CAAT-box. In addition, *pYCE1* and *pYCE2* possessed several *cis*-elements related to plant physiological regulation, such as light-responsive, auxin-responsive, salicylic acid-responsive, and ABA-responsive elements (**Supplementary Table S1**). The successfully cloned *pYCE1* and *pYCE2* will be utilized in subsequent functional assays to evaluate their potential to drive gene transcription in callus specifically.

### 3.3 *pYCE1* specifically and efficiently drives enhanced green fluorescent protein gene transcription in cassava callus

**Figure 2:**
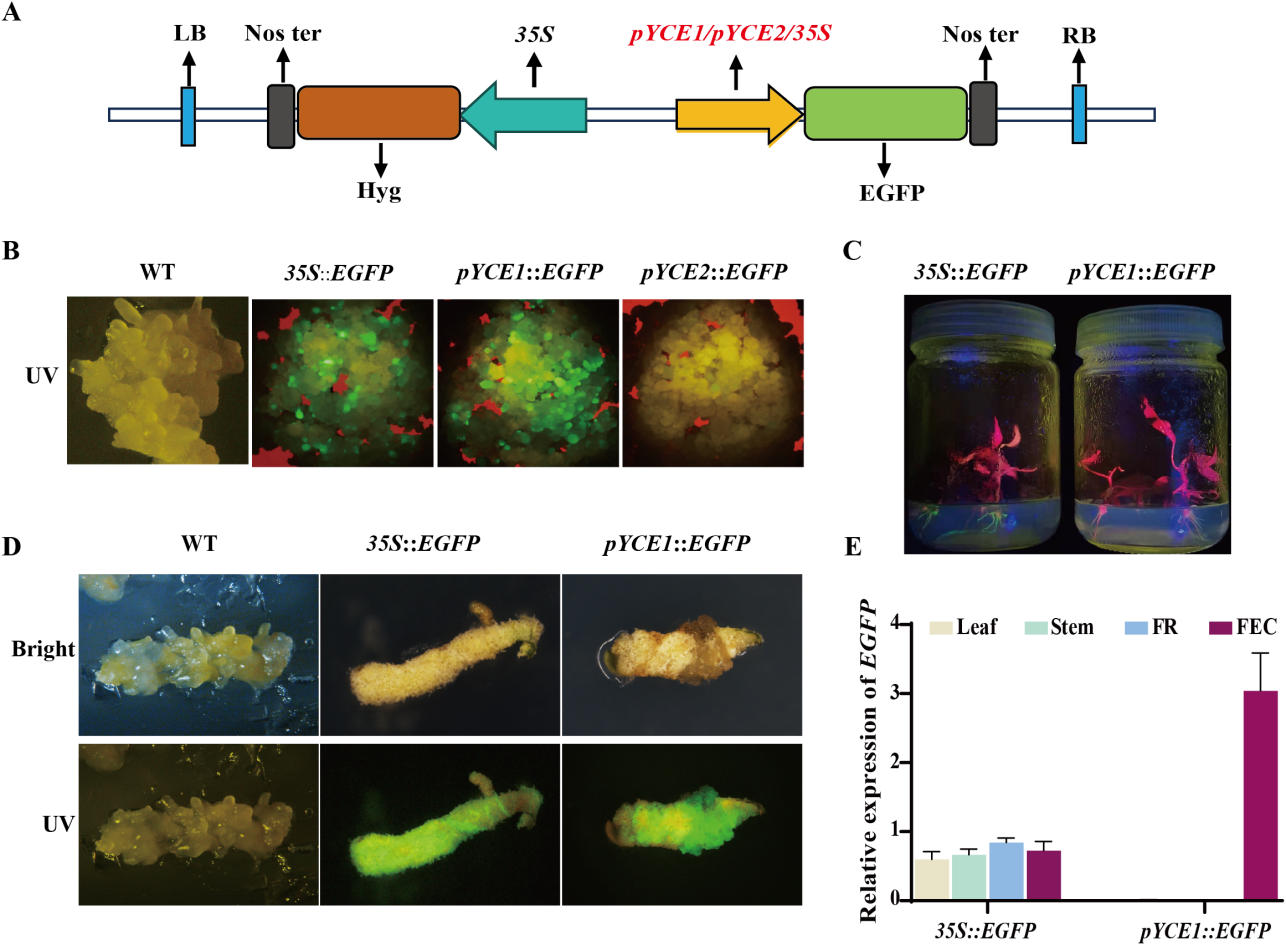
Identification of callus-specific Promoters. **(A)** *Promoter::EGFP* vector construct with the inserted promoter shown in red. **(B)** EGFP in callus tissues under UV light. WT: wild type. **(C)** EGFP in positive plants under UV light. **(D)** The transgenic positive plants were re-induced into callus tissue, and EGFP expression was observed under UV light. WT: wild type. **(E)** *EGFP* expression levels in different tissues of transgenic plants.

We constructed *pYCE1*::*EGFP* and *pYCE2*::*EGFP* vectors, with *35S*::*EGFP* as a control (**Figure 2A**), to assess the specificity and efficiency of *pYCE1* and *pYCE2* in driving gene transcription in cassava callus. These constructs were then transformed into FECs mediated by *Agrobacterium*. Following the fluorescence microscopy observation of the transgenic callus, only *pYCE1*::*EGFP* and *35S*::*EGFP* exhibited visible green fluorescence. The fluorescence intensity in *pYCE1*::*EGFP* was significantly higher than that in *35S*::*EGFP*. Therefore, *pYCE1* possesses stronger promoter activity and can drive higher efficiently transcription of *EGFP* in callus. On the contrary, no visible green fluorescence was observed in *pYCE2*::*EGFP* (**Figure 2B**), which suggested that *pYCE2* has low activity, and its callus-specificity remains unverified.

Subsequently, the callus containing *pYCE1*::*EGFP* and *35S*::*EGFP* were induced to regenerate into shoots and plants, which were then confirmed by PCR assay (**Supplementary Figure S1B**). EGFP expression was observed under UV light. Due to the presence of chlorophyll in cassava leaves and stems, which interferes with the green fluorescence emitted by EGFP, fluorescence observation was primarily focused on the roots. In other tissues, *EGFP* expression was assessed using RT-qPCR. In the leaves, stems, and roots of *pYCE1*::*EGFP* positive plants, *EGFP* expression was nearly undetectable, and no green fluorescence was observed in the roots. By contrast, *35S*::*EGFP* plants exhibited strong green fluorescence in the roots, and *EGFP* expression was detected in all tissues (**Figures 2C, E**). When *pYCE1*::*EGFP* plants were re-induced to generate callus, green fluorescence re-emerged, with the fluorescence intensity in *pYCE1*::*EGFP* being higher than that in *35S*::*EGFP* (**Figure 2D**). These results indicated that *pYCE1* exhibits callus-specific expression and can efficiently drive *EGFP* transcription in callus.

### 3.4 *pYCE1* drives *Cas9* transcription and enhances gene editing efficiency

**Figure 3:**
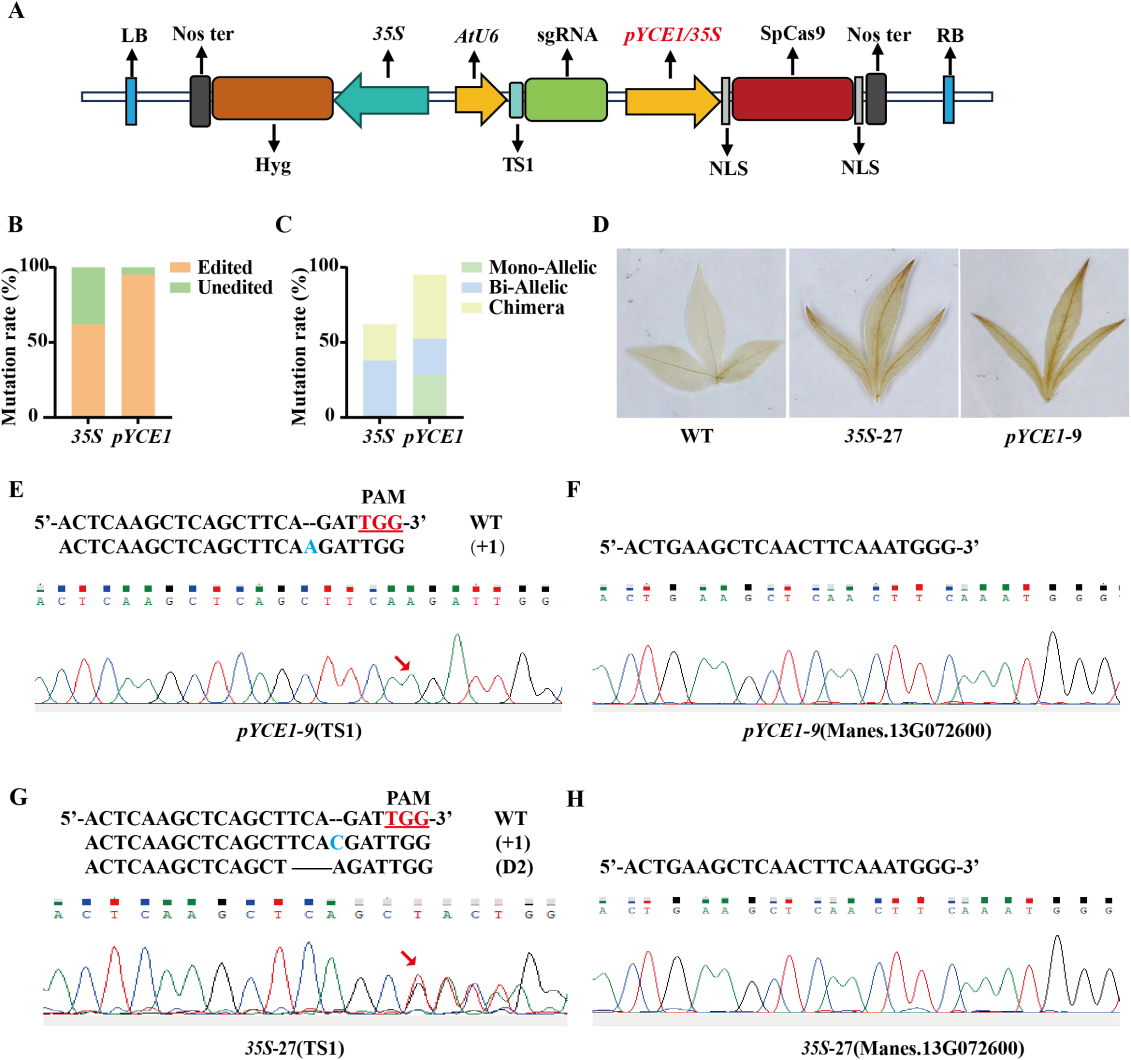
Gene editing efficiency comparison of *pYCE1*::Cas9 and *35S*::Cas9. **(A)** Structure of the editing vector with the promoter insertion site marked in red. TS1: Target sequence 1. **(B)**, **(C)** Statistics of editing efficiency and homozygous mutation rates in transgenic plants driven by *35S*::Cas9 and *pYCE1*::Cas9. **(D)** DAB staining for assessing H_2_O_2_ content in leaves of wild type, *pYCE1-9*, and *35S-27* lines. WT, wild type. **(E)**, **(G)** Sanger and Hi-TOM sequencing for identifying mutations at the target sequences in *pYCE1–9* and *35S-27* lines. The PAM sequence is underlined; +, insertion; D, deletion. **(F)**, **(H)** Sanger sequencing was used to identify whether off-target effects were present.

We replaced *35S* with *pYCE1* to drive *Cas9* transcription for improving the gene editing efficiency of the CRISPR/Cas9 system in cassava. Using *MePOD3* (Manes.12G072300) as the target, we constructed two vectors *35S::Cas9-MePOD3* and *pYCE1::Cas9-MePOD3* (**Figure 3A**). These vectors were introduced into the FECs of cassava cultivar SC8 via *Agrobacterium*-mediated transformation. After rooting screening and molecular identification, 29 positive plants with *35S*::*Cas9* and 21 positive plants with *pYCE1*::*Cas9* were obtained (**Supplementary Figures S2A, B**). Hi-Tom sequencing was used to determine the mutant types of these positive plants. Among the 29 positive plants with *35S*::*Cas9*, 18 plants were edited, with a mutation rate of 62.07% (**Figure 3B**, **Table 1**). Among them, 11 plants were homozygotes, all of which were biallelic homozygotes, no monoallelic homozygous was detected, and the remaining 7 plants were chimeras (**Table 1**). On the contrary, among the 21 positive plants with *pYCE1*::*Cas9*, 20 plants were edited, which resulted in a high mutation rate of 95.24% (**Figure 3B**, **Table 1**). Among them, 11 plants were homozygotes, with a homozygous mutation rate of 52.38%, including 5 monoallelic and 6 biallelic homozygotes; meanwhile, the remaining 9 plants were chimeras (**Figure 3C**, **Table 1**).

**Table 1:** Statistics of editing efficiency and homozygous mutation rates in transgenic plants.

Positive strain	Number	Mutation rate	Homozygous Mono-Allelic	Homozygous Bi-Allelic	Chimera
*35S*::Cas9-MePOD3	29	62.07% (18/29)	0.00%(0/29)	37.93%(11/29)	24.14%(7/29)
*pYCE1*::Cas9-MePOD3	21	95.24% (20/21)	28.57%(6/21)	23.81%(5/21)	42.86%(9/21)

We perform Hi-Tom sequencing to detect the mutant type. For *pYCE1–*9 line, a single A base was inserted at the TS1 site, which led to a monoallelic homozygous mutant. By contrast, the *35S-*27 line exhibited two editing types at the TS1 site, which resulted in insertion of an A base and deletion of TC bases, therefore, a biallelic homozygous mutation occurred (**Supplementary Table S3**). Subsequently, Sanger sequencing confirmed the insertion of an A base at the TS1 site in the *pYCE1-*9 (**Figure 3E**), while the *35S*-27 exhibited overlapping peaks at the TS1 site, which suggested the presence of multiple mutation types (**Figure 3G**). In addition, to further assess the stability of the modified CRISPR/Cas9 editing system, we conducted Sanger sequencing analysis on the top-priority predicted off-target sites (identified via CRISPR P2.0) for each of the targeted loci (TS1). Results confirmed no detectable off-target mutations at the sites (**Figures 3F, H**). The *MePOD3* gene encodes a peroxidase that effectively decomposes H_2_O_2_. DAB (3,3’-diaminobenzidine) staining of leaves from *pYCE1–*9 and *35S*-27 lines revealed a significant increase in H_2_O_2_ contents compared with the wild type (**Figure 3D**). These results demonstrated that the *Cas9* transcription driven by *pYCE1* not only significantly enhances the overall mutation rate but also facilitates the generation of homozygous mutant in cassava.

### 3.5 *pYCE1*-driven *Cas9* enables efficient dual-gene editing

**Figure 4:**
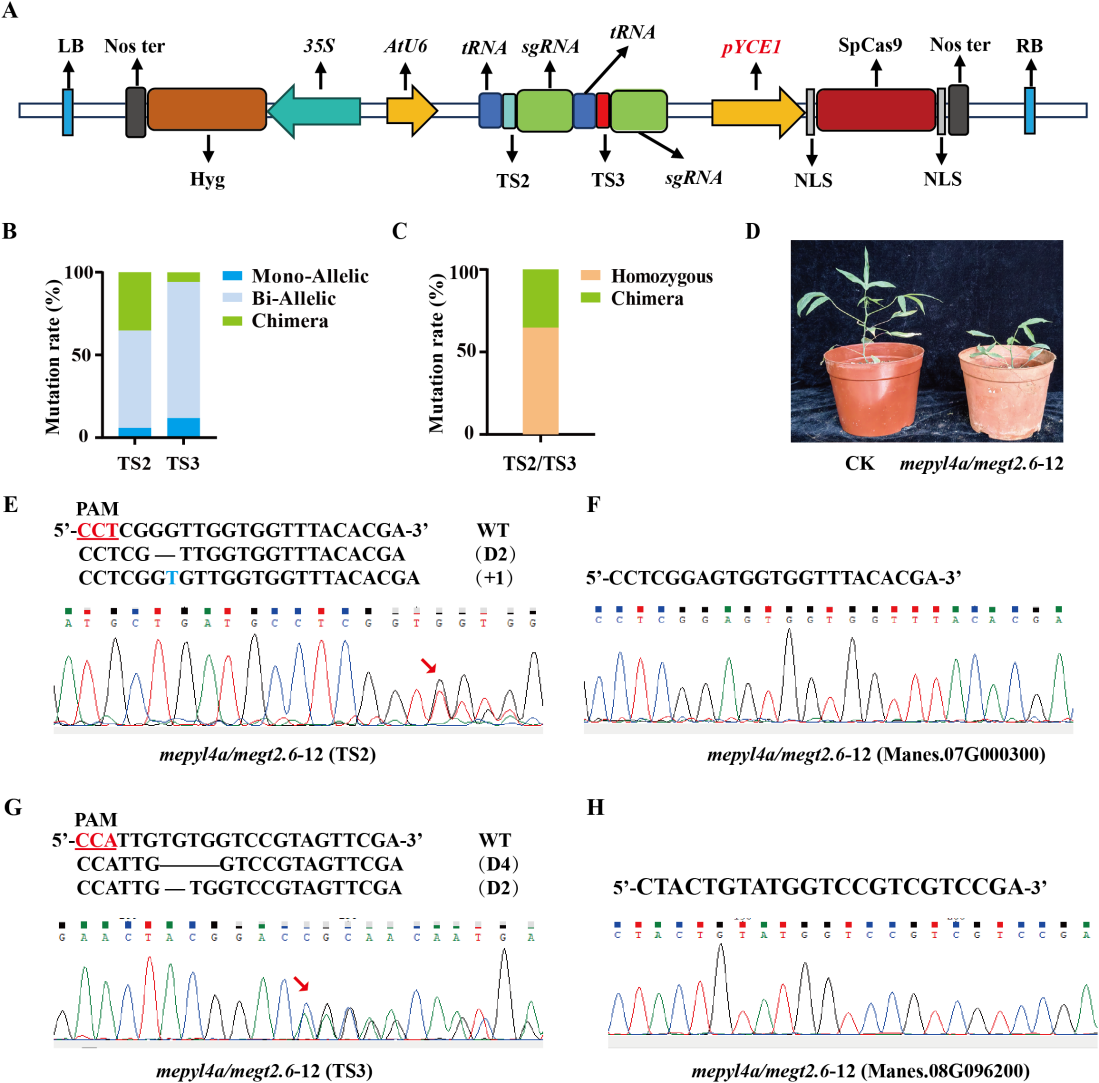
Efficiency of *pYCE1*::Cas9 in dual-gene editing. **(A)** Schematic map of the dual-gene editing vector. The red region indicates the inserted *pYCE1*; TS2, target sequence 2; TS3, target sequence 3. **(B)**, **(C)** Mutation rates and homozygous mutation statistics at TS2, TS3, and the TS2/TS3 dual-target sites. **(D)** Phenotype of wild type and *mepyl4a/megt2.6–12* mutant. **(E)**, **(G)** Sanger sequencing and Hi-TOM sequencing for identifying mutation types. The PAM sequence is underlined; +, insertion; D, deletion. **(F)**, **(H)** Sanger sequencing was used to identify whether off-target effects were present.

We constructed the dual-gene editing vector *pYCE1::Cas9-MeGT2.6+MePYL4a* (**Figure 4A**) to further evaluate the effectiveness of *pYCE1*-driven *Cas9* in dual-gene editing. This vector contains two target sites, namely, TS2 and TS3, which target the transcription factor *MeGT2.6 (Manes.08G021500)* (Bao et al., 2024) and the ABA receptor protein gene *MePYL4a (Manes.09G110800), respectively*. After transformation into FECs with the dual-gene editing vector, 17 positive plants were obtained (**Supplementary Figure S2C**). Hi-Tom sequencing revealed that TS2 and TS3 sites had a mutant rate of 100% among the 17 positive plants (**Figure 4C**, **Table 2**). At TS2 site, 11 plants exhibited homozygous mutation, with a homozygous mutation rate of 64.71%. These plants included 1 monoallelic homozygous mutant and 10 biallelic homozygous mutants. At the TS3 site, 16 plants were homozygous mutants, with a homozygous mutation rate of 94.12%; these plants included 2 monoallelic homozygous mutants and 14 biallelic homozygous mutants (**Figure 4B**, **Table 2**). In addition, off-target analysis was conducted for the sites that show high similarity to TS2 and TS3. After PCR amplification, Sanger sequencing of the sites in *Manes.07G000300* and *Manes.08G96200* revealed no base mutations, indicating the absence of off-target effects (**Figures 4F, H**).

**Table 2:** Mutation rates and homozygous mutation statistics at TS2, TS3.

Positive strain	Number	Mutation rate	Homozygous	Chimera
TS2	17	100% (17/17)	64.71% (11/17)	35.29% (6/17)
TS3	17	100% (17/17)	94.12% (16/17)	5.88% (1/17)
TS2/3	17	100% (17/17)	64.71% (11/17)	35.29% (6/17)

The *gt2.6/pyl4a-12* line was a homozygous mutant, with its TS2 (*MeGT2.6*) site having biallelic homozygous mutations involving the deletion of 2 base (GG) and insertion 1 bases (T); meanwhile, its TS3 (*MePYL4a*) site also had biallelic homozygous mutations involving the deletion of 4 bases (TGTG) and 2 bases (TG) (**Figures 4E, G**, **Supplementary Table S4, S5**). Summarizing the editing types of the 17 positive plants shows that 11 plants had homozygous mutations at TS2 and TS3 sites, with a dual-gene homozygous mutation rate of 64.71% (**Figure 4C**). Phenotypic analysis of the *gt2.6/pyl4a-12* edited plants revealed that, compared to the control (CK), the *gt2.6/pyl4a-12* plants were significantly shorter (**Figure 4D**). This phenotype is consistent with that of the previously reported *gt2.6* mutants (Bao et al., 2024), indicating that the editing system functions properly in dual-gene editing. These results indicated that *pYCE1::Cas9* not only achieves high editing efficiency but also enables a high proportion of dual-gene homozygous mutant.

## 4 Discussion

In modern molecular biology and plant biotechnology research, the rational selection and utilization of different types of promoters are crucial for achieving precise gene expression regulation. Based on their expression characteristics, promoters can be categorized into three types: inducible promoters, constitutive promoters, and tissue-specific promoters (Villao-Uzho et al., 2023). Constitutive promoters, such as the *35S* promoter and the ubiquitin gene (*UBI*) promoter, are widely used in transgenic technologies due to their ability to drive gene expression uniformly across various plant tissues (Xing et al., 2014). The broad application of these promoters lies in their provision of sustained and stable expression patterns, making them suitable for genes that need to be continuously expressed throughout all stages of plant growth and development. However, this indiscriminate expression can sometimes result in energy waste and may adversely affect plant physiology, especially when the expressed protein possesses biological activity or toxicity (Li et al., 2018). In contrast, tissue-specific promoters activate gene expression only in particular tissues, such as root-specific promoters, making them ideal for research that requires precise spatial control of gene expression (Li et al., 2013). *GELPs* play a crucial role in plants, contributing significantly to plant growth and development, responses to abiotic stress, as well as the formation of pollen and anthers (Cenci et al., 2022). In this study, we identified a GDSL-type esterase/lipase (*GELP*) gene, *YCE1*, which exhibits high and specific expression in cassava callus. Moreover, its promoter, *pYCE1*, demonstrated callus-specific expression, similar to the callus-specific promoter *CSP* identified in rice, which has been shown to drive the specific expression of the GUS reporter gene in callus (Zhou et al., 2020). By utilizing this promoter, we successfully achieved efficient expression of *EGFP* in cassava callus.

The efficiency of CRISPR/Cas9-mediated editing can vary significantly across species. Selecting appropriate promoter to drive *Cas9* transcription is crucial to improving editing efficiency and increasing the homozygous mutation rate. Enhancing *Cas9* transcription is important to achieve high gene editing efficiency within the CRISPR/Cas9 system (Ma et al., 2015). The callus-specific *DMC1* promoter in maize, the strong *Ubiquitin* promoter in cotton, and the egg cell-specific *EC2.1* promoter in *Arabidopsis* have all been utilized to enhance *Cas9* transcription; this enhancement significantly increased gene editing efficiency in their respective systems (Feng et al., 2018; Li et al., 2022; Wang et al., 2015). In cassava, the *35S* promoter has been used to drive *Cas9* transcription, which resulted in a homozygous mutation rate of less than one-third. By contrast, using the *YAO* promoter, which is preferentially expressed in tissues undergoing active cell division, increased the homozygous mutation rate to 75%; this augmentation greatly enhanced gene editing efficiency (Wang et al., 2022). However, *YAO* promoter is an exogenous promoter without FEC tissue specificity. It may lead to excessive Cas9 accumulation in non-target tissues, which potentially increases the risk of off-target effects or changes editing types across different generations. This study identified a callus-specific promoter, *pYCE1*, which was used to drive *Cas9* transcription. It achieved 95.24% mutation rates and a homozygous mutation rate of 52.38% in single-gene editing, respectively. In the dual-gene editing approach, the stability of sgRNA expression was enhanced by linking each sgRNA with a tRNA (**Figure 4A**). This strategy resulted in a 100% mutation rate and a 64.71% homozygous mutation rate, achieving the highest multi-gene editing efficiency ever reported in cassava. Given the success of this method in dual-gene editing, it holds significant reference value for future studies involving three or more gene edits. Additionally, this editing system demonstrated strong stability, with no off-target effects observed at the three targeted loci in this study. Meanwhile, it reduced Cas9 accumulation in non-callus tissues. Overall, *pYCE1* efficiently and specifically drives *Cas9* transcription in callus. This phenomenon significantly enhances homozygous gene editing efficiency, especially in dual-gene editing.

The secondary structure of sgRNA also plays a critical role in gene editing efficiency. A more stable stem-loop structure enhances the binding affinity of sgRNA to Cas9, which improves cleavage efficiency (Huszár et al., 2023). The sgRNA structures formed by different target site sequences vary and affect Cas9 cleavage efficiency (Huang et al., 2023; Ren et al., 2023). In this study, three target sites also showed varied editing efficiencies, which ranged from 62.07% to 100%; this result further emphasized the importance of target site selection (**Supplementary Figure S3**). In addition, the expression of sgRNA is essential, and the use of different promoters to drive sgRNA across species results in varying gene editing efficiencies (Develtere et al., 2024). In this study, the *AtU6* promoter was used to drive single and tandem sgRNAs, both achieving high mutation rates. However, exploring the use of endogenous sgRNA promoter with strong activity will be beneficial for further improving single/multi-gene homozygous mutation rates in cassava. The combined application of the *pYCE1* with an appropriate cassava *U6* promoter is expected to further enhance gene editing efficiency. This approach will advance gene function studies and facilitate the creation of new germplasm in cassava.

## Data Availability

The original contributions presented in the study are included in the article/**Supplementary Material**. Further inquiries can be directed to the corresponding author/s.
