# Supplementary material for: Improving gene editing of CRISPR/Cas9 using the callus-specific promoter pYCE1 in cassava (Manihot esculenta Crantz)
Source: Front Plant Sci. 2025 May 20;16:1600438. doi: 10.3389/fpls.2025.1600438 (PMC12130021; doi:10.3389/fpls.2025.1600438)
Supplement: Supplementary file 1 [file DataSheet1.docx]

Supplementary Material

# Supplementary Figures and Tables

## Supplementary Figures


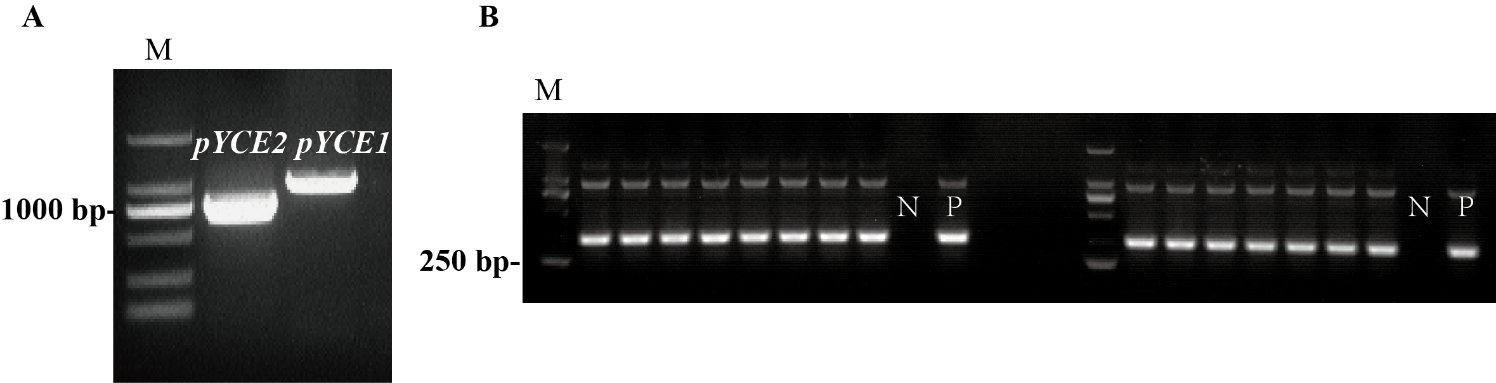


Supplemental Figure S1

Identification of the tissue specificity of *pYCE1.* **(A)** Cloning of *pYCE1* and *pYCE2*. **(B)** Identification of positive plants for *35S::EGFP* and *pYCE1::EGFP*. M: DNA Marker, N: Negative, P: Positive.


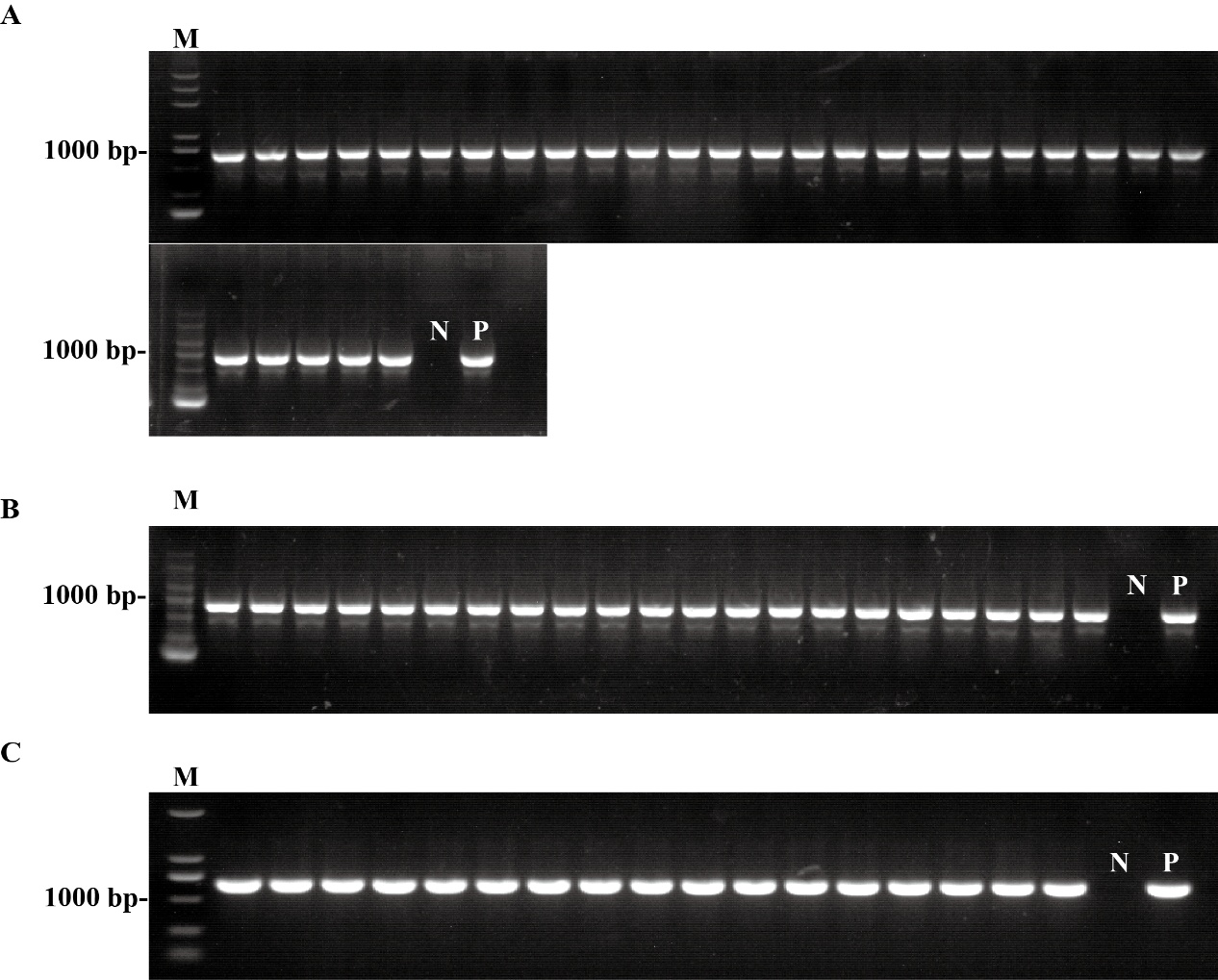


Supplemental Figure S2

Identification gene-edited positive plants by PCR. **(A)** Identification of positive lines for *35S*::Cas9-MePOD3. **(B)** Identification of positive lines for *pYCE1*::Cas9-MePOD3. **(C)** Identification of positive lines for *pYCE1*::Cas9-MeGT2.6+MePYL4a. M: DNA Marker. N: Negative; P: Positive.


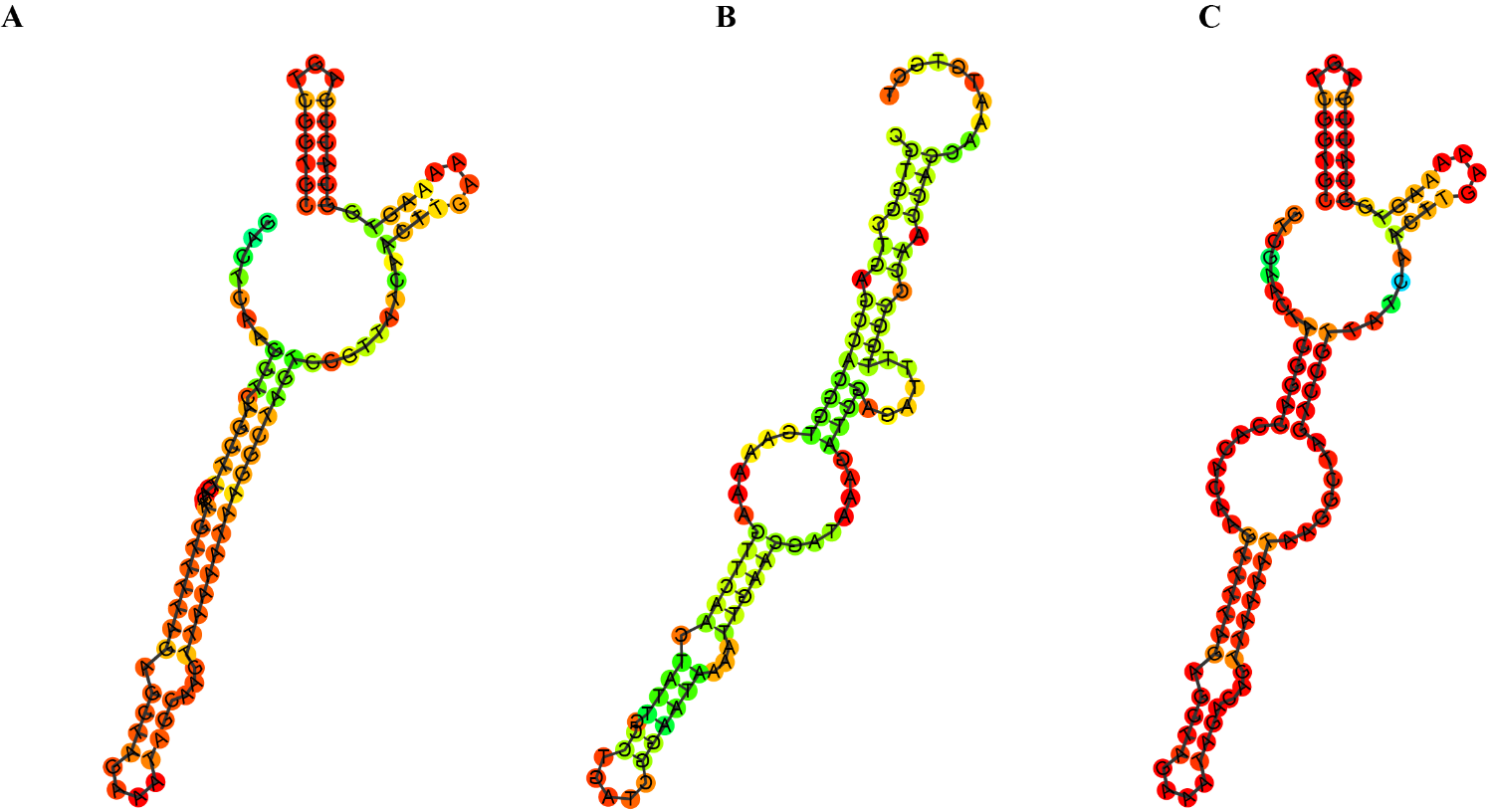


Supplemental Figure 3

Secondary structures of sgRNA formed by target sites TS1, TS2, and TS3. **(A)** TS1-sgRNA. **(B)** TS2-sgRNA. **(C)** TS3-sgRNA.

*pYCE1* promoter sequence:

ACACGTCGCTATACATCCCCAAGGTATGAGACTAGAGGTGAGCAGTATTCAGTTTAAACCGAGCGAATCGAATCGATTTGAAAATTTGATTCGGTTTTTTATACATTTCGGTTTGGTTCGATTTTTAATTTTAAAAATTTCAGTTATTTCGATTCGATTCGATTTTGATCAGAAAAAAACCGAAAAAACTGAACCGAACCGATTAGTAATAATAATATATTTTTCAATAATATAGAGAAATTAAATCATATTAAAATTAAAATATTTTAATTAAATTTTAAAATATTAAAAATAAAATATAAAAAATAAAAAATTATTAAAAATCAAAACCGATCAAACCGAATCGAATCGAATCGAATCAGACCGGTTCGATTCGATTCGATTTCTGACCAAAATCGGTTCGATTCGGTTTTCATAAACACTAAAATTTCAGTTTTCGGTTTATTCGATTCGGTTCGGTTTTGAACCGAACCGACCGAATGCTCACCCCTAATGAGACTGTGGGCAGTGTGAGAAAATATGTGGTATATATATATTAAATTATTAATTATTTATAATTTAAATAATAAAATAATAATAAATTCATATAAAATCGCATTTATATTAGTAGATATAATTAATTACCATTCAACCAAAATATTTAATTTTTCTTTTTTTTTTCAAAAAAATATTTAATTTTCTTGTGATTGTTTCCAAGCTCTTTATAGGATAAGTATGGGATTGGTTGAATGAGGATAGCTAAATACCTATTGCATTGCTCATTTGACATTAAAGAAGATGGGCAAAAAGAAGTTCAGAACCTAGAATTTTTCTTGCCAAAGGGAAGATGAGAGAAATTAGAACTATCAAGTGGCTGACTAGCTAAGCAGCAGAAAATGGATAACATAATCAAGATTGAAGAAAAGAAAAATTCCCTCTTTTTGTTAAGAACCTAGTAGGTTAAAAATGAAAAAAGAAAGAAGAAATGCATATGATTTTAGGAATGTACATTAACTTTTAGGGTAAAGAATAGGGTTGACAAGATGAAAAATATTAATACCTGGTGAAGGTTTTTTGCCTTGTTTTTGGGTACACATGCATCAGAAAGTAATTACTGCAGGAGGAAGAGAACAGCTGAATGCATTTACTTCTACTCAACCACCCACCTTACCAGCTTGCCTATTAAATCTCCCTTGATCGCACTCACACTTCACACAACTTCACCATAACAGTGCCCATCACCTGTTTGTTTCTTTGTCTCGCTGACCCAG
