# Supplementary figures and images for: Improving gene editing of CRISPR/Cas9 using the callus-specific promoter pYCE1 in cassava (Manihot esculenta Crantz)
Source: Front Plant Sci. 2025 May 20;16:1600438. doi: 10.3389/fpls.2025.1600438 (PMC12130021; doi:10.3389/fpls.2025.1600438)

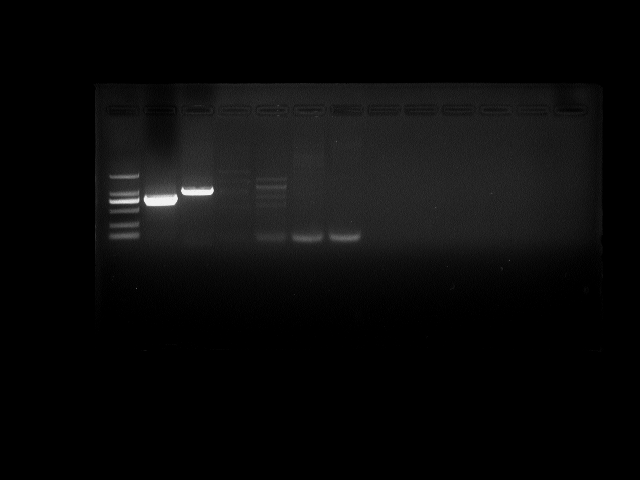

Supplement: Supplementary file 3 [file Image1.tif]

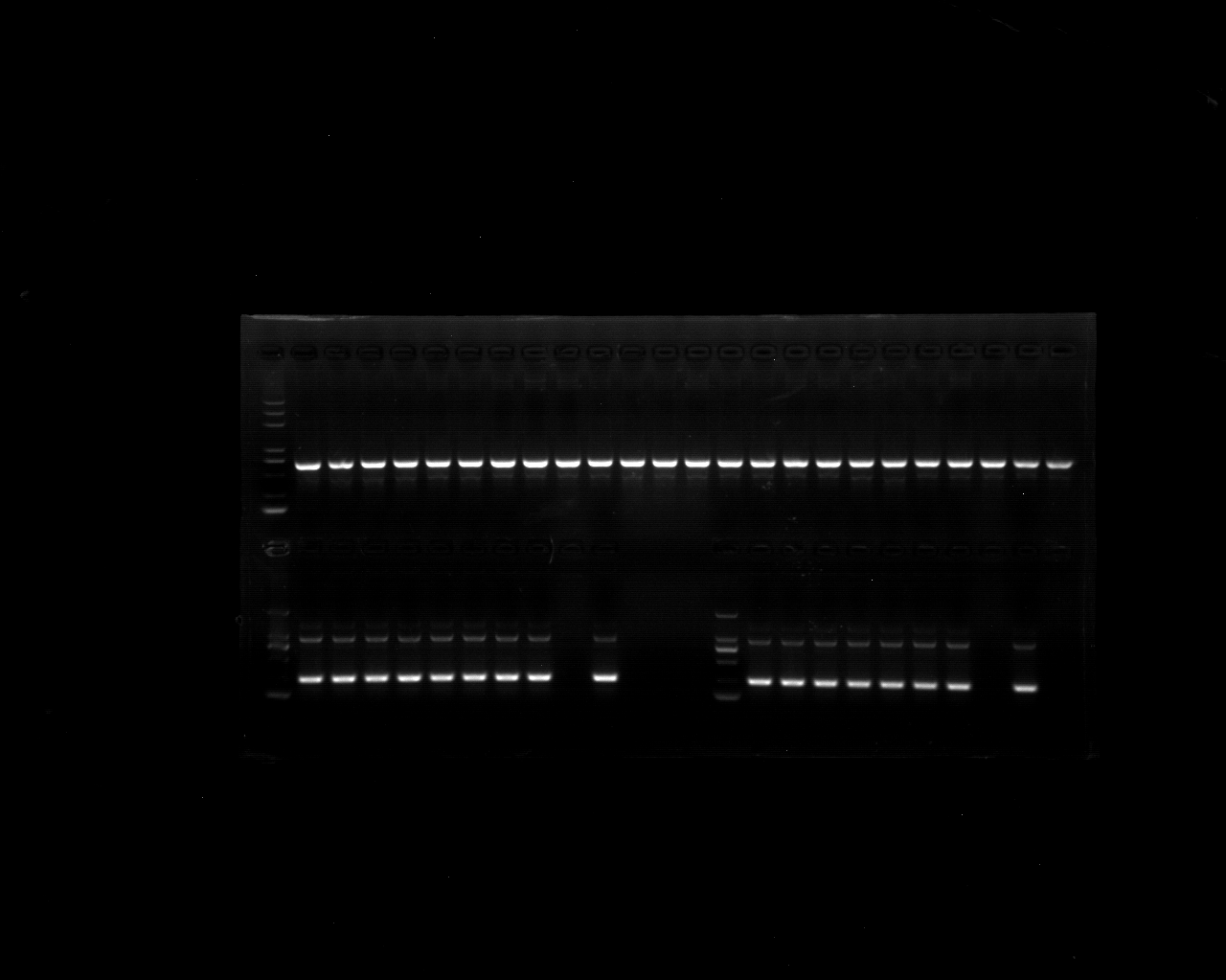

Supplement: Supplementary file 4 [file Image2.tif]

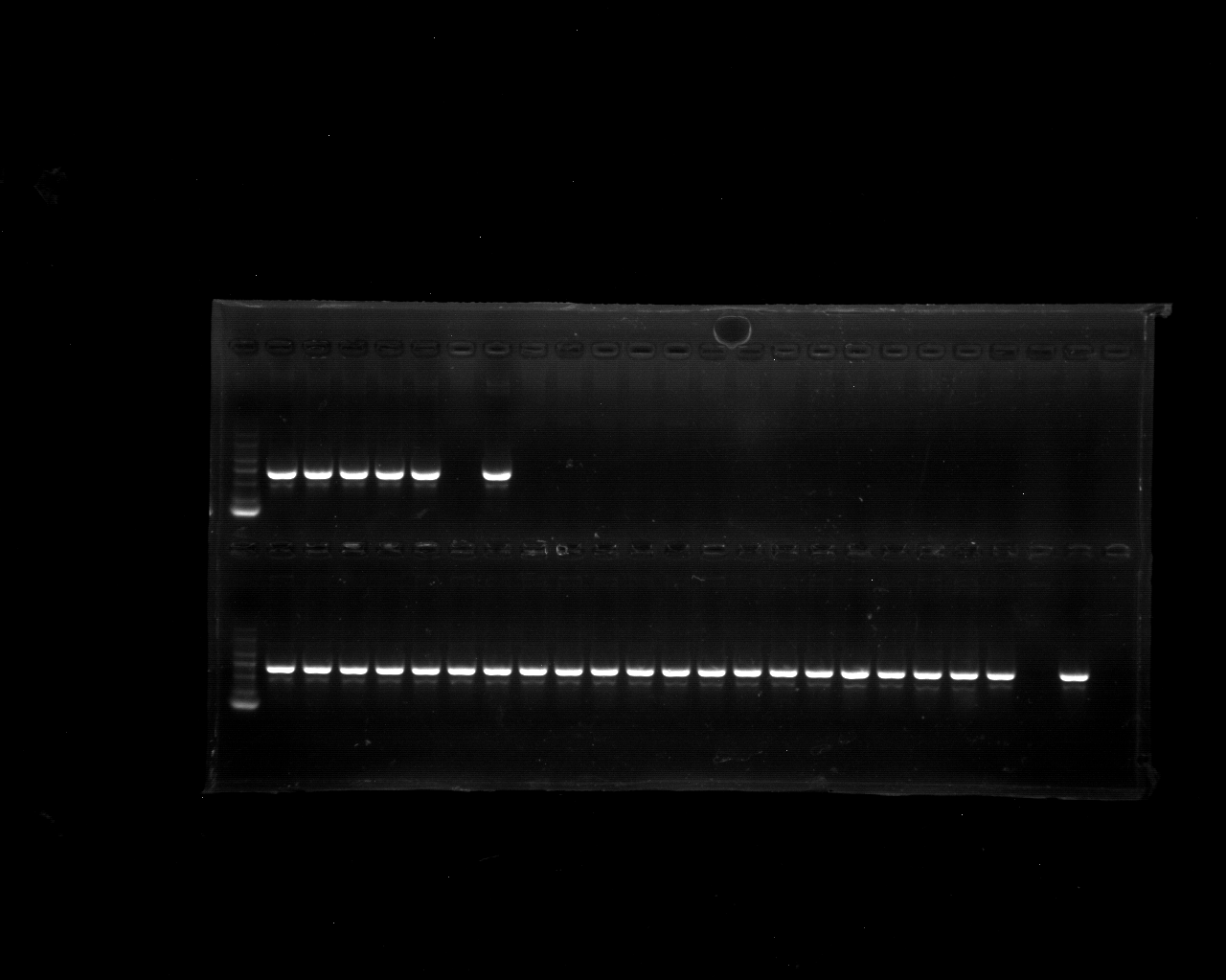

Supplement: Supplementary file 5 [file Image3.tif]

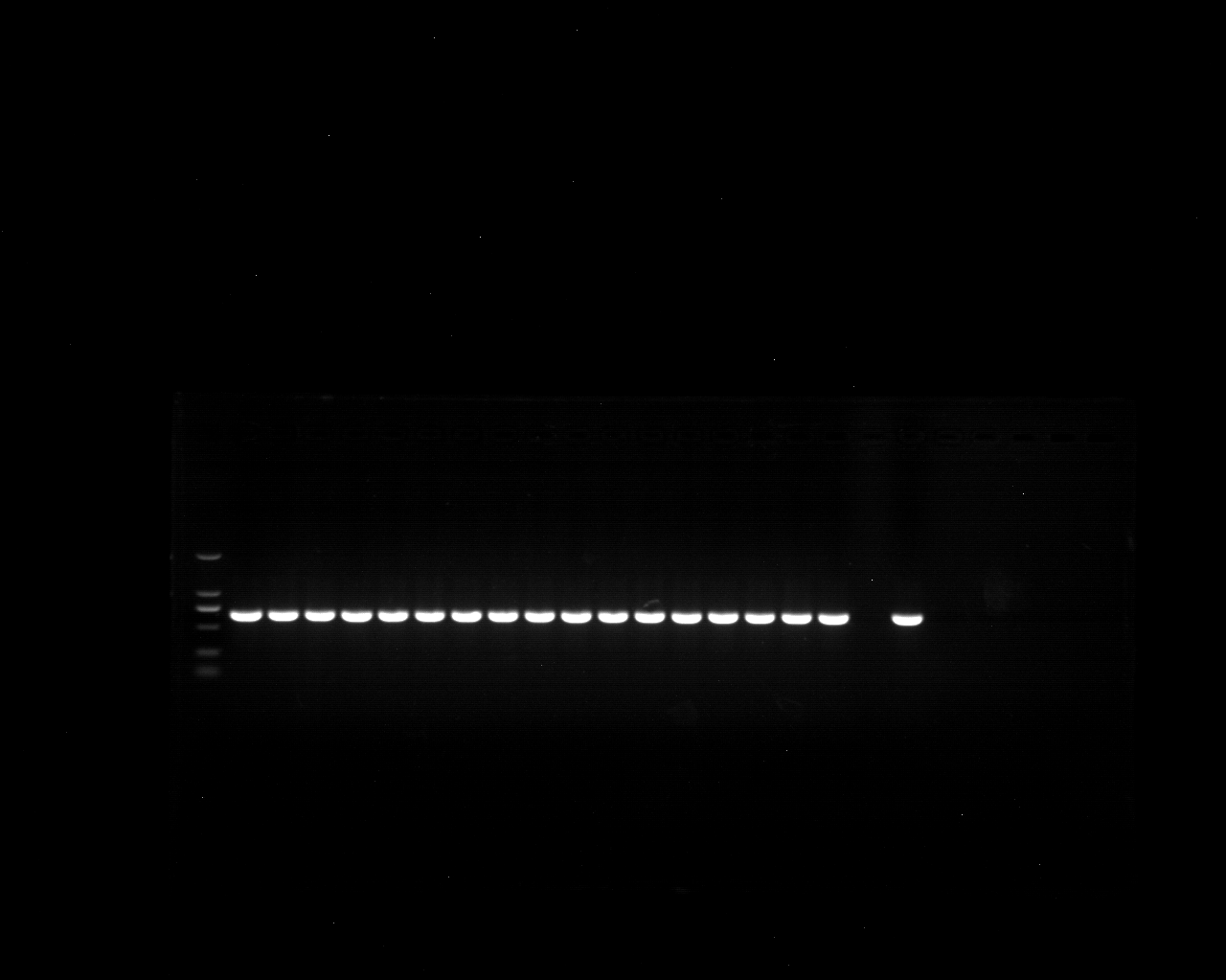

Supplement: Supplementary file 6 [file Image4.tif]
